# Supplementary material for: Non fire-adapted dry forest of Northwestern Madagascar: Escalating and devastating trends revealed by Landsat timeseries and GEDI lidar data
Source: PLoS One. 2024 Feb 20;19(2):e0290203. doi: 10.1371/journal.pone.0290203 (PMC10878523; doi:10.1371/journal.pone.0290203)
Supplement: S1 Table — (DOCX) [file pone.0290203.s001.docx]

**S1 Table. Kruskal-Wallis (chi-statistic and p-value) and Dunn tests (p-values) on plant area index, canopy cover, and canopy height across varying numbers of forest fires.**

| **Forest Attribute** | **Kruskal-Wallis (H; p)** | **0 fires vs 1 fires** | **0 fires vs 2 fires** | **0 fires vs 3 fires** | **1 fire vs 2 fires** | **1 fire vs 3 fires** | **2 fires vs 3 fires** |
| --- | --- | --- | --- | --- | --- | --- | --- |
| Plant area index | 310.67; <0.001 | < 0.001 | < 0.001 | < 0.001 | 0.005 | < 0.001 | 0.007 |
| Canopy cover | 310.75; <0.001 | < 0.001 | < 0.001 | < 0.001 | 0.005 | < 0.001 | 0.007 |
| Canopy height | 129.92; <0.001 | < 0.001 | < 0.001 | < 0.001 | n.s. | n.s. | n.s. |

n.s. denotes ‘not significant’, indicating that the p-value was above the threshold for statistical significance: p > 0.05.
