# Supplementary material for: Non fire-adapted dry forest of Northwestern Madagascar: Escalating and devastating trends revealed by Landsat timeseries and GEDI lidar data
Source: PLoS One. 2024 Feb 20;19(2):e0290203. doi: 10.1371/journal.pone.0290203 (PMC10878523; doi:10.1371/journal.pone.0290203)
Supplement: S2 Table — Also included in the model are two random variables: the park’s management zone (community, buffer, or service) and season (dry season May-October vs. wet season November-April). (DOCX) [file pone.0290203.s002.docx]

# S2 Table. Linear mixed models testing for the effects of the number of fires (n_fires) and the number of months since the last fire (n_months) on three attributes of forest structure: plant area index, canopy cover, and canopy height. Also included in the model are two random variables: the park’s management zone (community, buffer, or service) and season (dry season May-October vs. wet season November-April).

| **Forest Attribute** | **Term** | **Effect** | **Estimate** | **Std. Err.** | **df** | **p** | **Var.** |
| --- | --- | --- | --- | --- | --- | --- | --- |
| Plant area index | intercept | fixed | 1.0804 | 0.2482 | 3.4054 | 0.0172 * | - |
|  | n_fires | fixed | -0.2003 | 0.0692 | 521.4599 | 0.003 ** | - |
|  | n_months | fixed | 0.0037 | 0.0036 | 358.6113 | 0.306 | - |
|  | zone | random | - | - | - | - | 0.0464 |
|  | season | random | - | - | - | - | 0.0394 |
| Canopy cover | intercept | fixed | 0.3843 | 0.0469 | 3.765 | 0.0016 ** | - |
|  | n_fires | fixed | -0.077 | 0.0155 | 169.2 | 0.0000 *** | - |
|  | n_months | fixed | 0.0005 | 0.0008 | 158.5 | 0.5771 | - |
|  | zone | random | - | - | - | - | 0.0004 |
|  | season | random | - | - | - | - | 0.0015 |
| Canopy height | intercept | fixed | 13.3658 | 1.0123 | 4.3751 | 0.0001 *** | - |
|  | n_fires | fixed | -0.9589 | 0.3346 | 389.4674 | 0.0044 ** | - |
|  | n_months | fixed | -0.0409 | 0.0174 | 252.0357 | 0.0199 * | - |
|  | zone | random | - | - | - | - | 0.438 |
|  | season | random | - | - | - | - | 0.5649 |

The terms ‘n_fires’ and ‘n_months’ represent the number of fires experienced and the number of months since the most recent fire respectively.
